# B7-H3 suppresses CD8^+^ T cell immunologic function through reprogramming glycolytic metabolism

**DOI:** 10.7150/jca.90819

**Published:** 2024-03-11

**Authors:** Yulu Wu, Wenzhe Han, Xiufa Tang, Jiyuan Liu, Zhiyong Guo, Zhangao Li, Chenchen Cai, Lin Que

**Affiliations:** State Key Laboratory of Oral Diseases & National Center for Stomatology & National Clinical Research Center for Oral Diseases, West China Hospital of Stomatology, Sichuan University, Chengdu 610041, Sichuan, China; Department of Oral and Maxillofacial Surgery, West China Hospital of Stomatology, Sichuan University, Chengdu 610041, Sichuan, China.

## Abstract

Malignant neoplasms pose a formidable threat to human well-being. Prior studies have documented the extensive expression of B7 homolog 3 (B7-H3 or CD276) across various tumors, affecting glucose metabolism. Yet, the link between metabolic modulation and immune responses remains largely unexplored. Our study reveals a significant association between B7-H3 expression and advanced tumor stages, lymph node metastasis, and tumor location in oral squamous cell carcinoma (OSCC). We further elucidate B7-H3's role in mediating glucose competition between cancer cells and CD8^+^ T cells. Through co-culturing tumor cells with flow cytometry-sorted CD8^+^ T cells, we measured glucose uptake and lactate secretion in both cell types. Additionally, we assessed interferon-gamma (IFN-γ) release and the immune and exhaustion status of CD8^+^ T cells. Our findings indicate that B7-H3 enhances glycolysis in OSCC and malignant melanoma, while simultaneously inhibiting CD8^+^ T cell glycolysis. Silencing B7-H3 led to increased IFN-γ secretion in co-cultures, highlighting its significant role in modulating CD8^+^ T cell functions within the tumor microenvironment and its impact on tumorigenicity. We also demonstrate that glycolysis inhibition can be mitigated by exogenous glucose supplementation. Mechanistically, our study suggests B7-H3's influence on metabolism might be mediated through the phosphoinositide3-kinase (PI3K)/ protein kinase B (Akt)/ mammalian target of rapamycin (mTOR) signaling pathway. This research unveils how B7-H3 affects immune functions via metabolic reprogramming.

## Introduction

Malignant tumors present a significant threat to human health, with head and neck malignancies ranking as the sixth most common cancer 1. Among these, OSCC constitutes over 90% of oral and maxillofacial malignancies 2-4, while melanoma is responsible for more than 70% of skin cancer-related deaths due to its high metastatic potential. Immunotherapies targeting immune checkpoints, such as programmed cell death protein 1 (PD-1)/ programmed cell death 1 ligand 1 (PD-L1)/ programmed cell death 1 ligand 2 (PD-L2), have shown promising prospects for treating OSCC and melanoma 5, 6. The B7 family of immune checkpoints, including PD-L1, B7-H3, and B7 homolog 4 (B7-H4), plays a crucial role in tumor immunotherapies. These proteins are abnormally expressed in tumor cells and tumor-associated immune cells, contributing to tumor immune evasion and patient prognosis 7. Immune checkpoint inhibitors targeting PD-1 and/or PD-L1 have demonstrated their efficacy in clinical studies and are considered a second-line treatment option for recurrent and metastatic OSCC 8. Combination therapy using longevity assurance gene 1 (LAG-1) inhibitors and PD-1 inhibitors is approved by the United States Food and Drug Administration as first-line treatment for patients with metastatic melanoma 6.

B7-H3 is another member of the B7 family, which is highly expressed in various solid tumors, such as breast cancer, bladder cancer, pancreatic cancer, and esophageal cancer 9-15. It exhibits both immune co-stimulatory and co-inhibitory effects, making it an important target for immune checkpoint therapy 16, 17. Studies show that it connects to tumor growth, metastasis, chemotherapy sensitivity, apoptosis, and angiogenesis 18-21. Notably, B7-H3 has been found to regulate glucose metabolism. Studies found that B7-H3 increases the rate of extracellular acidification and lactate production in metastatic melanoma, reflecting increased levels of glycolysis 22. In addition, inhibition of B7-H3 in breast cancer enhances sensitivity to targeted therapies by inhibiting glycolysis 23. In OSCC, B7-H3 is highly expressed and highly glycosylated, correlating with tumorigenicity, tumor stage, and lymph node metastasis 24. This suggests that B7-H3 may influence tumor microenvironmental metabolic reprogramming.

The Warburg effect, characterized by increased glycolysis even in the presence of sufficient oxygen, is a hallmark of malignancies, providing energy for tumor growth, invasion, and metastasis 25, 26. Immune cells in the tumor microenvironment (TME) also undergo metabolic reprogramming, with effective T cells heavily relying on glycolysis for energy during proliferation and differentiation 27, 28. In contrast, regulatory T cells and myeloid-derived suppressor cells tend to use oxidative phosphorylation or fatty acid oxidation for energy 29, 30. This competition for energy might affect the immune functions of CD8^+^ T cells, promoting T cell functional exhaustion and immune escape 31-33

Based on this understanding, we hypothesize that B7-H3 might promote glycolysis and metabolic reprogramming in the TME, leading to energy competition between tumor cells and CD8^+^ T cells. This competition could impair the energy supply to CD8^+^ T cells, reducing their normal physiological functions. By focusing on OSCC and melanoma, this study aims to investigate the role of B7-H3 in tumor development, contributing to potential B7-H3-based targeted therapies and immunotherapies.

## Materials and Methods

### Patients and Specimens

OSCC and matched adjacent healthy tissue samples were obtained from the West China School of stomatology, Sichuan University, following stringent selection criteria. These criteria included patients who had undergone surgical removal of squamous cell carcinoma, as confirmed by pathological diagnosis. Exclusion criteria ruled out individuals who had received pre-operative treatments, such as chemotherapy and radiotherapy, or those with concurrent malignancies. The staging of these cases was conducted according to the 2017 American Joint Committee on Cancer (AJCC) TNM classification system, encompassing a total of 57 patients. This study received approval from the Ethics Board of the West China Hospital of Stomatology, Sichuan University.

### Cell lines, culture, and transfection conditions

The cancer cell lines were provided by State Key Laboratory of Oral Diseases and National Clinical Research Center for Oral Diseases, West China Hospital of Stomatology. These cell lines, including SCC7 and B16-F10, were rigorously tested and authenticated. They were subjected to infection with retroviral particles and corresponding control viruses obtained from Gene (GENE, Shanghai), designed to induce overexpression, silencing, or normal expression (vector control) of B7-H3. Selection of cells was achieved using 2 μM puromycin, with the concentration reduced to 1 μM upon the cessation of noticeable cell death, thereby establishing stable cell lines with either overexpressed or silenced B7-H3. We adopted the following nomenclature for these cell lines and their corresponding controls: Normal Control (NC), Overexpression Vector (OV), Silencing Vector (SV), and their respective empty vector controls infected by the corresponding negative virus: Overexpression-Empty Vector (O-EV) and Silencing-Empty Vector (S-EV).

Cells in the logarithmic growth phase from each tumor cell group were harvested and enzymatically dissociated into single cells using 0.25% trypsin (Hyclone, US), followed by mechanical disaggregation.

Spleens were collected from 6-8 week-old male C57BL/6 mice under sterile conditions, minced, and strained through a 200-mesh sieve to achieve a single-cell suspension. To prevent non-specific antibody binding, Fc receptors were blocked using FcR blocking reagent (Miltenyi, Germany). Subsequently, the cells were stained with anti-mouse CD3 antibodies (CD3e Monoclonal Antibody (145-2C11), PerCP-Cyanine5 (eBioscience, US)) and anti-mouse CD8 antibodies (CD8a Monoclonal Antibody (53-6.7), FITC (eBioscience, US)) to facilitate the flow cytometric sorting of CD8^+^ T cells.

### Glucose uptake assay

After stimulation, the cells were divided equally into 2 ml cell suspensions in the upper chamber with the tumor cells in the lower chamber in the Transwell system (Corning, US) placed in a 6-well plate. The Glucose-free RPMI-1640 culture medium (Gibco, US) was supplemented with 10% fetal bovine serum (FBS) (Gibco, US) and 1000 U/mL of interleukin-2 (IL-2) (PeproTech, US). The ratio of CD8^+^ T cells to tumor cells was 5:1. After co-culturing for 72 hours, both the cells and the supernatant were collected for biochemical assays.

Cells (10^4^ per well) were seeded in 96-well, and were adjusted to 50ul/well using glucose assay buffer in the Glucose Colorimetric/Fluorometric Assay Kit (Biovision, US). With other procedures following the instructions of the kit, absorbance was measured at 570 nm on an enzyme marker.

### Lactate production assay

Cells (10^4^ per well) were seeded in 96-well, and were adjusted to 50ul/well using lactate assay buffer in the Lactate Colourimetric/Fluorometric Assay Kit (Biovision, USA). Following the instructions of the kit, and absorbance was measured at 570 nm.

### Quantitative real-time PCR (qRT-PCR)

Total RNA was isolated from cultured cells using Trizol reagent (TAKARA, Dalian). To create corresponding cDNA, DNase-treated total RNA was subjected to an iScript cDNA Synthesis Kit (Bio-Rad, USA) on a CFX96 Real-Time System with a C1000 Touch Thermal Cycler (Bio-Rad) using the following protocol: 5 min at 95°C, 40 cycles of 10 seconds at 95°C and 30 seconds at 60°C, and finally a 65°C to 95°C ramp up to determine the melting curve. The relative amounts of mRNA were calculated using the comparative Ct method.

### Western blot analysis

Cancer cells were lysed in RIPA buffer (Invitrogen, US) at 4°C for 15 min and centrifuged at 10000 × g at 4°C for 15 min to obtain total protein lysates for immunoblot analysis. Cell lysates were separated by 10% SDS-PAGE (Beyotime, Shanghai) and transferred onto PVDF membranes (Millipore, US). Proteins were then subjected to immunoblot. Primary antibody incubation was performed at 4°C overnight, and secondary antibody incubation was performed at room temperature for 1 h. The immunoreactive blots were visualized using an enhanced chemiluminescence reagent.

Anti-B7-H3 and anti-GAPDH antibodies were purchased from Huabio; anti-mTOR antibodies, anti-p-PI3K p85 (phospho-Tyr458) antibodies were purchased from Biobyt; anti-p-mTOR (Ser2448), anti-PI3K, anti-Akt, anti- glucose transporter 1 (GLUT1), anti-hypoxia-inducible factor 1-α (HIF-α) antibodies were purchased from CST. Goat anti-rabbit IgG; anti-6-phosphofructo-2-kinase3 (PFKFB3) antibodies were from Abcam and rabbit anti-mouse IgG secondary antibodies were purchased from Beyotime.

### Xenograft tumor studies

The Xenograft tumor studies were performed in accordance with protocols approved by the Institutional Animal Care and Use Committees of University of West China Hospital of Stomatology, Sichuan University. 4-5 week male C57BL/6 and C3H mice were used in the experiment. Each mouse received a subcutaneous injection of 2×10^6^ cancer cells suspended in 0.2 ml of PBS (Gibco, US) into the right axilla (forelimb). Tumor diameter was measured using digital calipers every 2-3 days based on the rate of tumor growth. Tumor volume was calculated using the following formula: Volume (mm^3) = (W^2 × L) / 2, where W represents the smaller diameter and L represents the larger diameter of the tumor.

### Flow cytometry

Aseptically obtained tumor tissue was digested with Collagenase IV (Biofroxx, Germany) for 2 hours. After collecting single-cell suspension, tumor-infiltrating lymphocytes were separated from the suspension using the Mouse Tumor Infiltrating Lymphocyte Separation Kit (Solarbio, Beijing). Fc receptors were blocked using Mouse FcR Blocking Reagent (Miltenyi, Germany), and then primary antibodies were added for incubation in flow cytometry. CD279 (PD-1) Monoclonal Antibody (RMP1-30) PE, CD366 (TIM3) Monoclonal Antibody (RMT3-23) PE-Cyanine7, IFN gamma Monoclonal Antibody (XMG1.2) PE and Granzyme B Monoclonal Antibody (NGZB) PE-Cyanine7 were bought from eBioscience. After washing, the cells were analyzed using the Attune NxT Flow Cytometer (Invitrogen, USA).

### Elisa analysis

The method for CD8^+^ T cell sorting and co-culturing was the same as described previously. For the inhibition of CD8^+^ T cell glycolysis, AZ-PFKFB3-67 (MedChemExpress, USA) was used at the concentration of 5μM, 10 μM and 20μM respectively for 24h after cell sorting and for another 24 hours after glucose supplementation. The culture supernatant was collected, and the content of IFN-γ was measured using Mouse IFN-γ ELISA Kit (Elabscience, Wuhan).

### Immunocytofluorescence (IF)

Tissues were fixed with 4% paraformaldehyde (PFA), embedded in paraffin, and sliced into 4-μm thick sections, which were then deparaffinized with xylene and rehydrated in graded ethanol. For antigen retrieval, the sections were incubated in EDTA buffer. After blocked with 5% BSA (Beyotime, Shanghai) in PBS, the sections were incubated overnight at 4°C with primary antibodies targeting the following proteins: B7-H3 (Proteintech Group, US), HIF-1α (CST, US), GLUT1 (CST, US), PFKFB3 (Abcam, UK), Akt (CST, US), mTOR (Biobyt, UK), p-PI3K p85 (phospho-Tyr458, Biobyt, UK), p-Akt (phospho-S473, Santa Cruz, US), p-mTOR (Ser2448, CST, US). Cells were then washed and incubated goat anti-rabbit secondary antibody (Beyotime, Shanghai) and rabbit anti-mouse secondary antibody (Beyotime, Shanghai) for 50 min. Finally, the cells were washed and incubated with DAPI (Beyotime, Shanghai) for 5 min at room temperature. Fluorescence images were visualized with a fluorescence microscope (NIKON, Japan).

### Immunohistochemistry (IHC)

The methodology for processing paraffin-embedded sections was consistent with that described for immunofluorescence (IF). Sections were blocked with 5% goat serum in phosphate-buffered saline (PBS) and incubated overnight at 4°C with primary antibodies specifically targeting B7-H3 (Proteintech, US). Following this, sections were stained using the SPlink Detection Kit (ZSGB-BIO, Beijing), with chromogenic development achieved using a DAB Kit (ZSGB-BIO, Beijing). After a brief counterstain with hematoxylin, the sections were examined and imaged with an Olympus fluorescence microscope. The IHC staining of CD276 in OSCC samples was assessed based on the staining ratio and intensity. The proportion score was determined by the estimated fraction of tumor cells positive for staining (0 = none; 1 = less than 25%; 2 = 25-75%; 3 = more than 75%), while the intensity score was assessed by the average staining intensity in positive tumor cells (0 = none; 1 = weak; 2 = moderate; 3 = intense). The total protein presence was then quantified as the immunoreaction score, calculated by multiplying the proportion and intensity scores (ranging from 0-9). All slides underwent evaluation by two independent researchers, providing four scores in total, from which an average was calculated for subsequent analysis.

### Statistical analysis

Data were derived from three experimental repeats, unless specified otherwise, and expressed as mean values for statistical representation. Statistical analyses of clinical samples were performed using SPSS 13.0 software, employing Student's t-test and one-way ANOVA to identify statistical differences, with a significance threshold set at P=0.05. Statistical outcomes were presented as mean ± standard deviation (SD). For other experimental data, statistical significance was determined using GraphPad Prism software version 9.5.1, employing t-tests. Significance levels were denoted as *p < 0.05, **p < 0.01, ***p < 0.001, and ****p < 0.0001. GraphPad Prism software version 9.5.1 and FlowJo software version 10.8.1 were utilized for figure creation.

## Results

### B7-H3 Expression Correlates with Clinicopathological Parameters

We investigated the expression levels of B7-H3 in 57 primary OSCC samples (Table 1). We found that B7-H3 expression was significantly correlated with tumor stage, lymph node metastasis, and tumor location. However, there was no significant correlation observed with gender, age, or recurrence rates (Table 2).

### B7-H3 Mediates Competitive Suppression of CD8^+^ T Cell Glycolysis in Oral Squamous Cell Carcinoma and Malignant Melanoma

**Figure 1:**
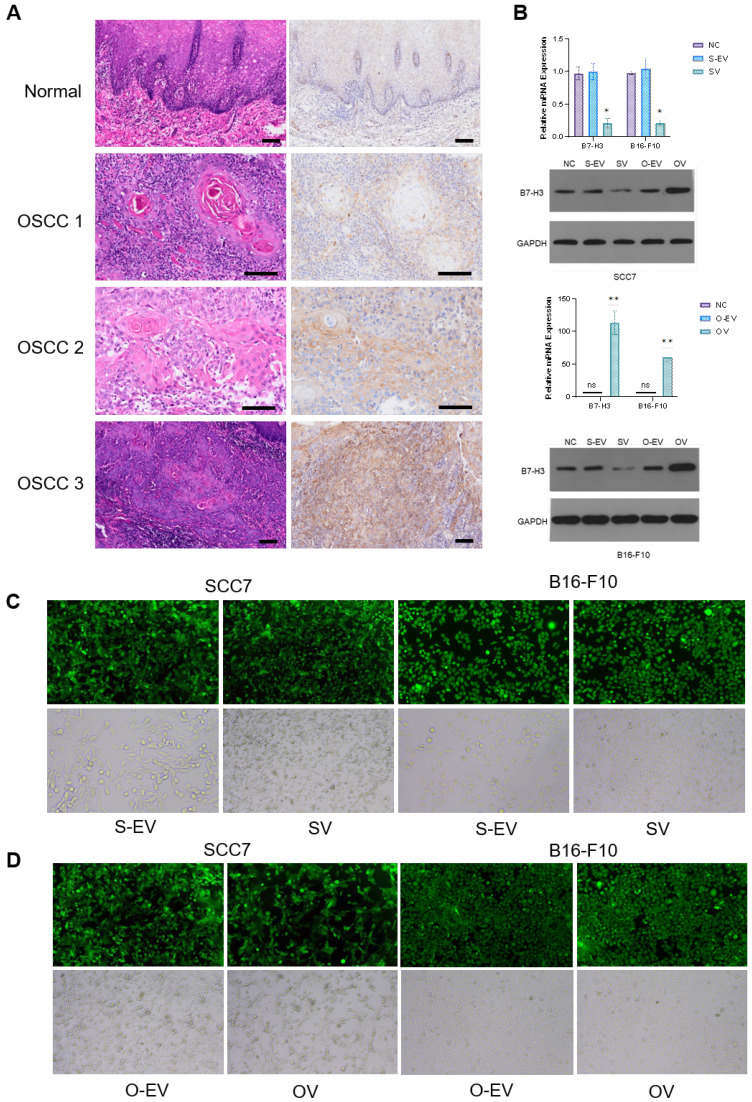
** Expression of B7-H3. (A)** Upregulation of B7-H3 in OSCC. B7-H3 protein expression was assessed by IHC (weak, moderate and intense expression in OSCC 1, OSCC 2 and OSCC 3 respectively, bar scale: 100μm). **(B)** Verification of lentiviral transfection in SCC7 and B16-F10 cell lines to establish stable cell lines with B7-H3 silencing and overexpression by RT-qPCR and western blot. **(C and D)** Observation of lentiviral transfection in SCC7 and B16-F10 cell lines under fluorescence microscopy. The transfection rates in both cell lines were more than 85%.

In this study, we employed lentiviral vectors to manipulate the expression of the B7-H3 in two cell lines: the oral squamous cell carcinoma cell line SCC7 and the malignant melanoma cell line B16-F10. The transfection efficiency was assessed using fluorescence microscopy to ensure that stable cell lines with B7-H3 silencing and overexpression were established (Figure 1C and D). The efficacy of the B7-H3 manipulation was confirmed through both RT-qPCR and Western Blot analysis (Figure 1B).

**Figure 2:**
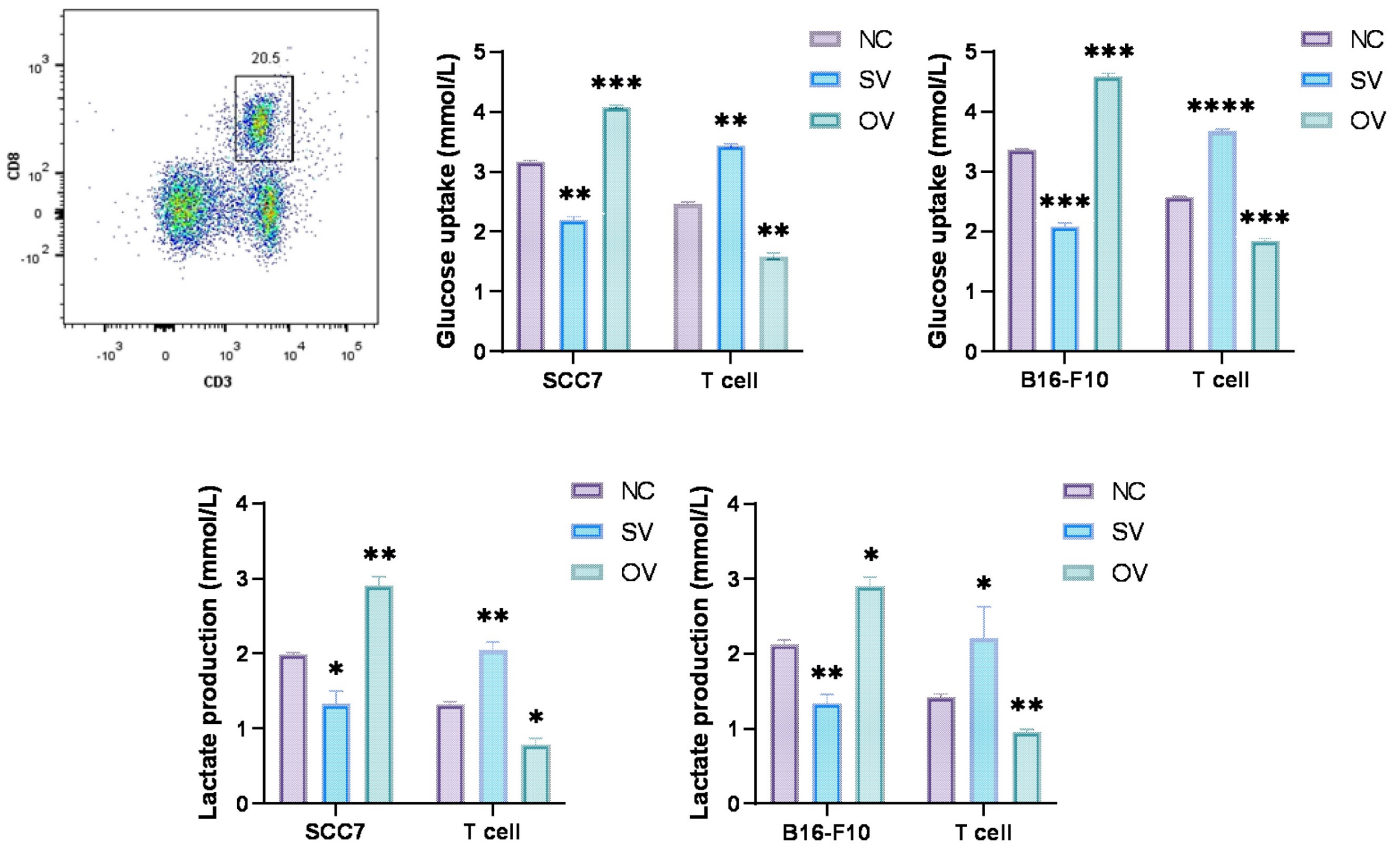
B7-H3 regulates aerobic glycolysis in SCC7 and B16-F10 cell lines. Glucose uptake and intracellular lactate production of tumor cells and T calls were detected in B7-H3 silenced vector and overexpression vector. A Flow cytometric sorting of mouse splenic CD8^+^ T cells. B-C B7-H3 regulates glucose uptake. D-E B7-H3 influences lactate production.

Numerous reports have indicated the involvement of B7-H3 in glucose metabolism regulation 2, 34. Prior investigations have demonstrated that tumor cells in murine sarcomas can hinder T cell glucose uptake through metabolic pathways 31. To investigate the implications of B7-H3 on tumor development, we established SCC7 and B16-F10 transplant mouse models with different B7-H3 expression and collected tumor tissues from these tumor-bearing mice and isolated CD8^+^ T cells from the mouse spleen using flow cytometry (Figure 2A). Subsequent to isolation, these CD8^+^ T cells were co-cultured with SCC7 and B16-F10 tumor cells that had been subjected to B7-H3 silencing or overexpression. Glucose uptake and intracellular lactate secretion of both tumor cells and T cells in the co-culture system were measured (Figure 2B-E). The results collectively suggest that B7-H3 can promote glycolysis in oral squamous cell carcinoma and malignant melanoma, while concurrently inhibiting glycolysis in CD8^+^ T cells, thereby implying the existence of a glycolysis competition between tumor cells and CD8^+^ T cells.

### Interplay of Glucose Metabolism Competition on T Cell Status and Immune Functions

T cells constitute a crucial component of the tumor microenvironment, and their acquisition of specific immune functions, particularly when confronted with external stimuli such as inflammation and tumors, necessitates substantial energy support 27. Since B7-H3 can enhance glycolysis in SCC7 and B16-F10 cells and indirectly impede the glycolytic activity of CD8^+^ T cells in the co-culture system, we postulated that there may be corresponding alterations in T cell function. Previous studies have highlighted that CD8^+^ T cells in the tumor microenvironment frequently manifest a state of exhaustion, characterized by diminished effector functions, including reduced cytokine production such as IL-2 and IFN-γ 35 and the upregulation of co-inhibitory molecules on the cell surface, notably PD-1, T cell immunoglobulin and mucin domain-containing protein 3 (Tim-3), and Lag-3 36.

**Figure 3:**
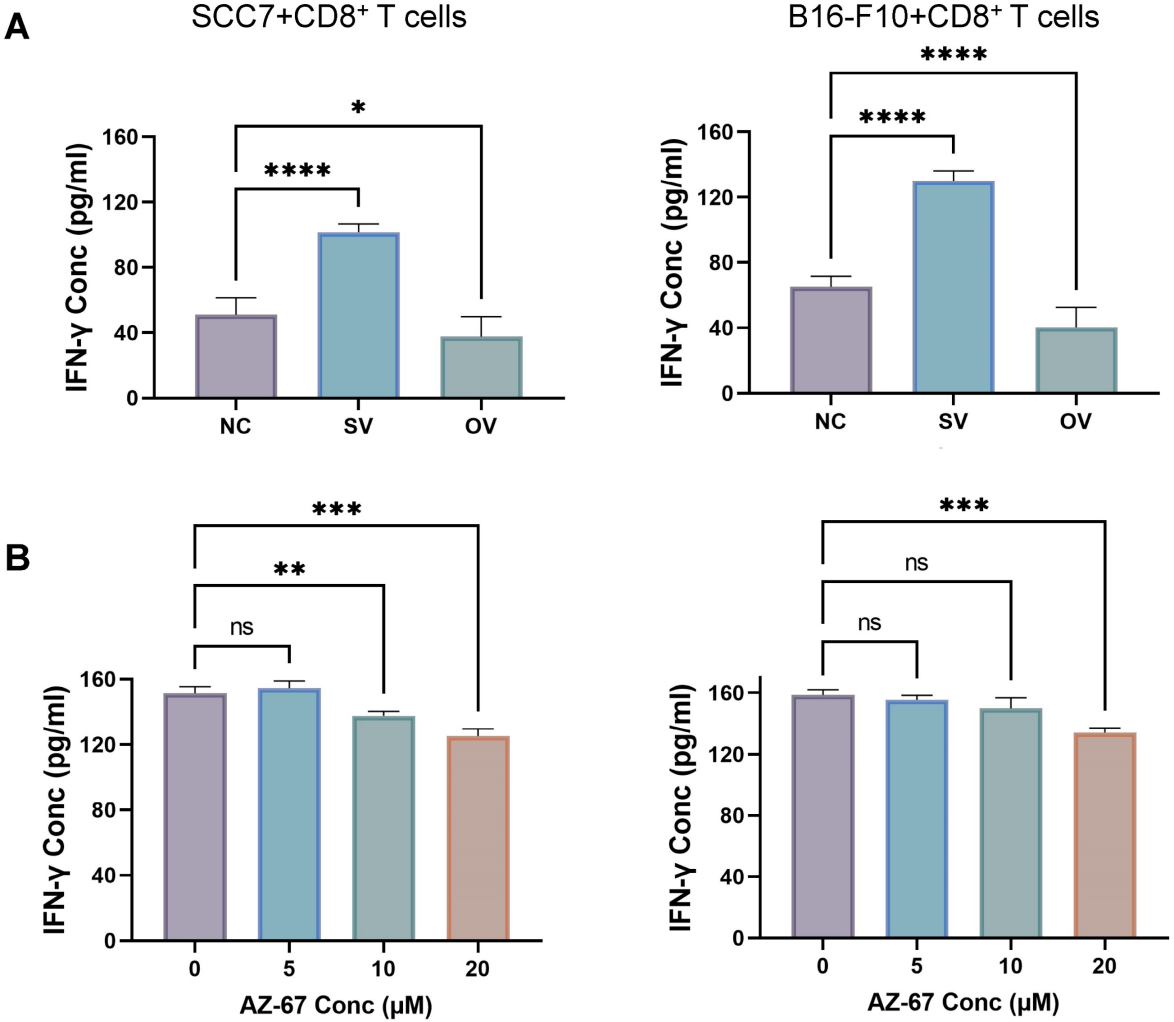
**(A)** B7-H3 regulates the secretion of IFN-γ of CD8^+^ T cells in the co-culture system, detected by ELASA assay. **(B)** Glycolysis inhibition can be mitigated by exogenous glucose supplementation. IFN-γ production was inhibited after incubation with the glycolysis inhibitor for 24 hours (left) and was mitigated after glucose supplementation (right).

**Figure 4:**
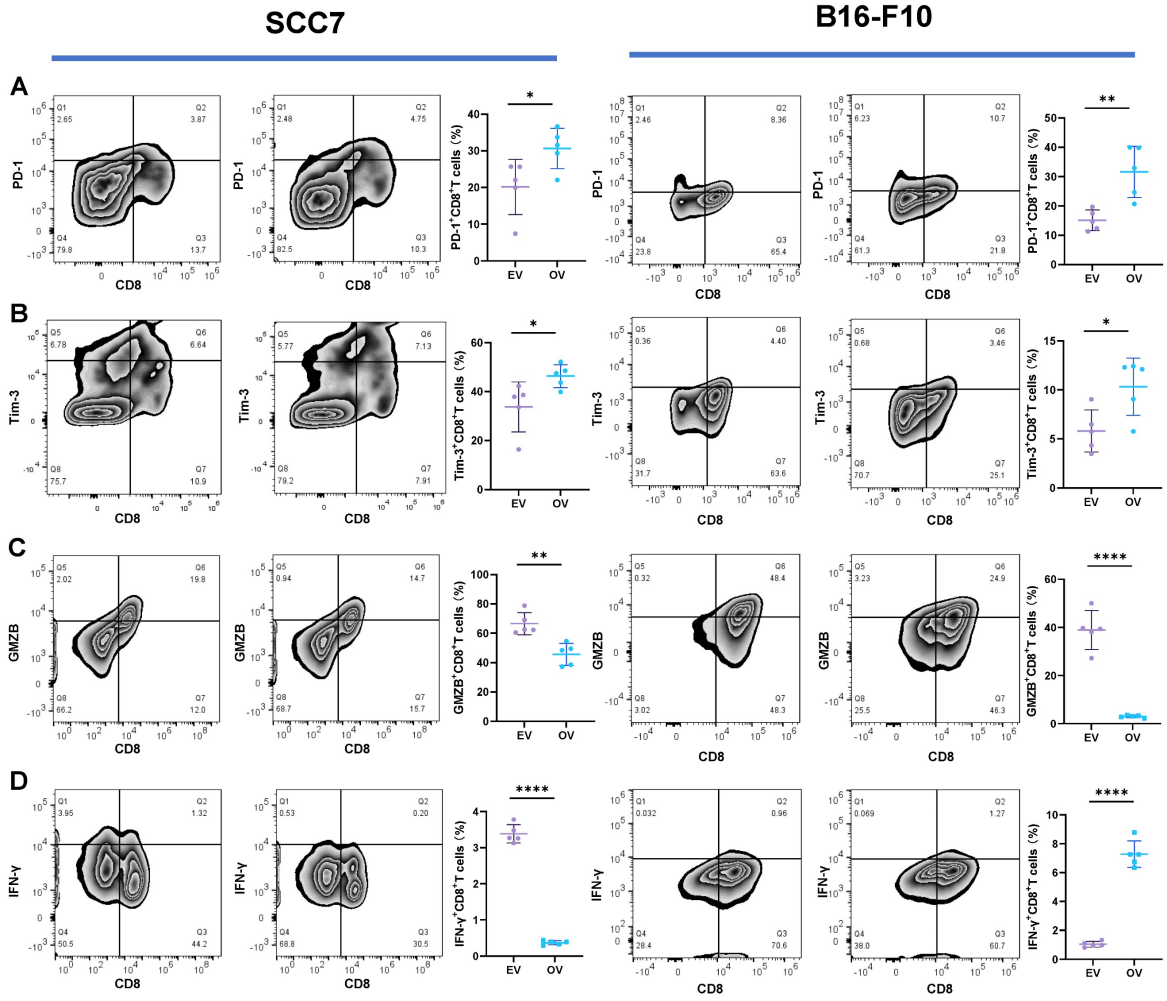
(A) Expression of PD-1 on tumor-infiltrating CD8^+^ T cells in tumor grafts. **(B)** Expression of Tim-3 on tumor-infiltrating CD8^+^ T cells in tumor grafts. **(C)** Expression of GZMB on tumor-infiltrating CD8^+^ T cells in tumor grafts. **(D)** Expression of IFN-γ on tumor-infiltrating CD8^+^ T cells in tumor grafts.

We employed the ELISA technique to assess the secretion of IFN-γ from T cells in the co-culture system (Figure 3A). Notably, CD8^+^ T cells from the B7-H3 silenced group exhibited increased secretion of IFN-γ. Furthermore, we discovered that the suppression of T cell function induced by glycolysis inhibition at a concentration of 10 μM could be reversed with the addition of exogenous glucose (Figure 3B). Subsequently, we evaluated the functional status of CD8^+^ T cells by analyzing intracellular IFN-γ and granzyme B (GZMB) production and evaluated CD8^+^ T cell exhaustion using the expression of PD-1 and Tim-3 on the cell surface in tumor-infiltrating lymphocytes (TILs) using flow cytometry (Figure 4). The overall increased proportion of PD-1^+^ and Tim-3^+^ CD8^+^ T cells and decreased proportion of IFN-γ^+^ and GZMB^+^ CD8^+^ T cells indicate that the overexpression of B7-H3 is associated with increased exhaustion markers and reduced effector molecule production in CD8^+^ T cells. Interestingly, the proportion of IFN-γ^+^ CD8^+^ T cells was elevated in the B16-F10 OV group. We speculate that such results are due to the difference in detection between ELISA and flow cytometry, which detect secreted and membrane-bound IFN-γ, respectively 37, and that membrane-bound IFN-γ grows significantly on the surface of B16-F10 cells in response to certain unknown factors. This suggests that B7-H3 may play a significant role in regulating CD8^+^ T cell functions in the tumor microenvironment, impacting immune responses against OSCC and melanoma. Further exploration of the intricate interplay between B7-H3, glucose metabolism, and CD8^+^ T cell function may provide valuable insights for developing novel immunotherapeutic strategies for cancer treatment.

### Enhanced Tumorigenicity in the Subcutaneous Xenograft Mouse Model by B7-H3 Overexpression

**Figure 5:**
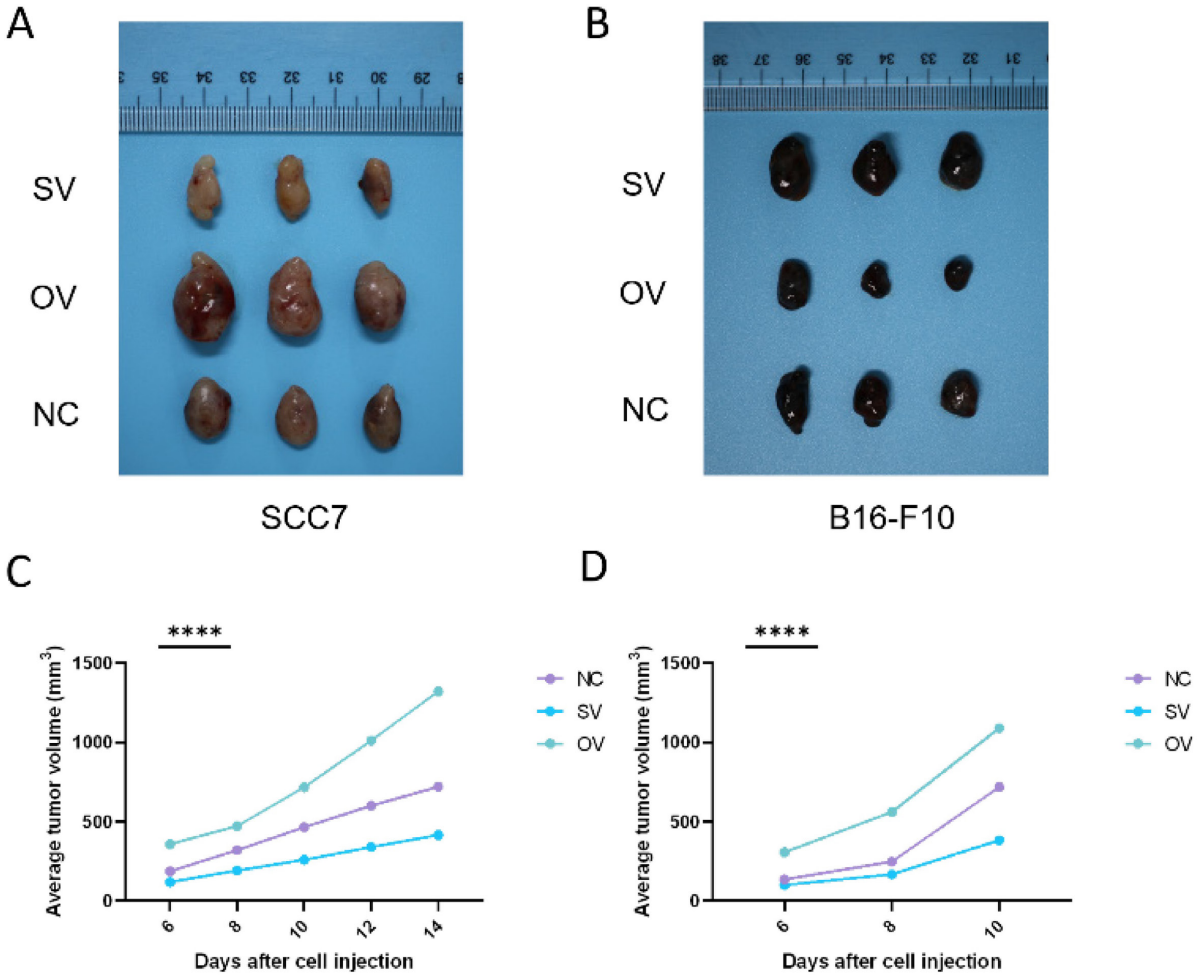
B7-H3 promotes tumorigenesis in OSCC and melanoma in subcutaneous xenograft mouse models. The tumor tissue was acquired on day 14 and 10 respectively and tumor volume was taken every two days.

In the subcutaneous xenograft mouse model, tumor samples derived from the SCC7 cell line were collected on the 14th day, while those from the B16-F10 cell line were harvested on the 10th day, as they exhibited faster growth. In both the SCC7 and B16-F10 cell lines, high expression of B7-H3 was related to enhanced tumorigenicity (Figure 5).

### Regulation of HIF-1α Expression by B7-H3 via the PI3K/Akt/mTOR Signaling Pathway

We have provided evidence of the competitive glucose utilization between tumor cells and CD8^+^ T cells, wherein the regulatory role of the B7-H3 molecule results in the suppression of CD8^+^ T cell immune function. Nevertheless, the precise molecular mechanisms underlying this phenomenon remain elusive. HIF-1α as a key player in cancer metabolism reprogramming, has been closely associated with aerobic glycolysis. Prior investigations have reported that B7-H3 governs the expression of HIF-1α through the PI3K/Akt/mTOR pathway 24.

**Figure 6:**
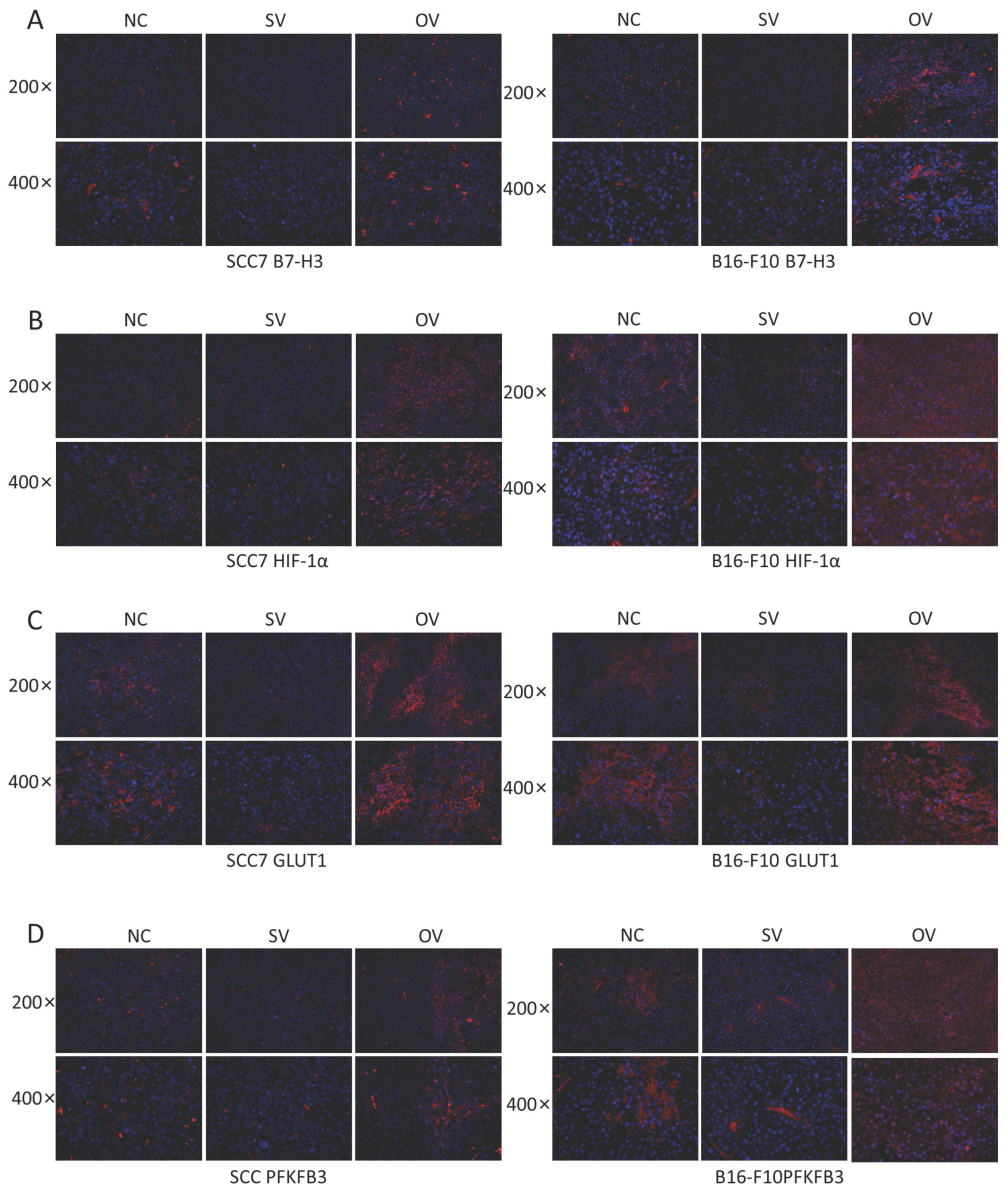
**(A)** Immunofluorescence was used to verify B7-H3 protein expression levels of different groups in mouse tumor tissues. **(B-D)** B7-H3 promotes HIF-1α, GLUT1 and PFKFB3 expression.

**Figure 7:**
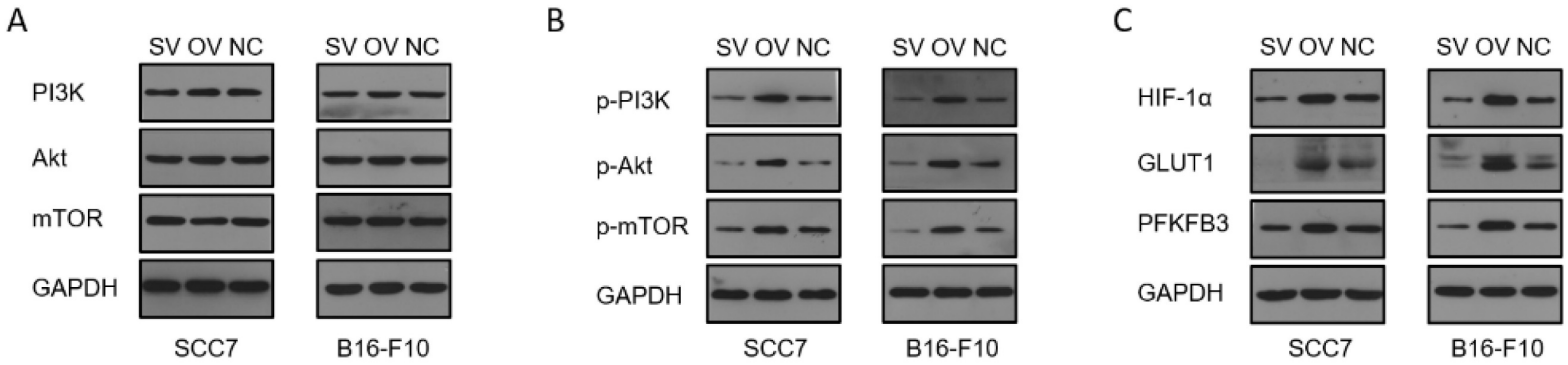
Regulation of B7-H3 on PI3K/Akt/mTOR pathway, HIF-1α, and downstream proteins by western blot. **(A)** B7-H3 silencing/overexpression does not affect the protein levels of PI3K, Akt, and mTOR in SCC7 and B16-F10 cells. **(B)** B7-H3 influences the levels of phosphorylated proteins p-PI3K, p-Akt, and p-mTOR in SCC7 and B16-F10 cells. **(C)** B7-H3 enhances the expression of HIF-1α and its downstream glycolysis-related proteins GLUT1 and PFKFB3.

To elucidate the potential regulatory role of B7-H3 in modulating HIF-1α expression through the PI3K/Akt/mTOR signaling pathway and its impact on downstream glycolysis-related proteins, we conducted immunofluorescence staining on tumor tissues collected from xenograft mouse models. The results revealed significant influence of B7-H3 on the fluorescence intensity of B7-H3, HIF-1α, GLUT1, and PFKFB3 (Figure 6). Western blot results demonstrated that the expression levels of PI3K, Akt, and mTOR were unaffected by varying levels of B7-H3 in both SCC7 and B16-F10 cell lines (Figure 7A). Nonetheless, B7-H3 overexpression resulted in the increased phosphorylation of these proteins (Figure 7B), subsequently leading to enhanced expression of HIF-1α and its downstream glycolysis-related proteins, GLUT1 and PFKFB3 (Figure 7C). Based on these experimental findings, we propose a hypothesis that B7-H3 might exert regulatory control over HIF-1α expression through the phosphorylation of the PI3K/Akt/mTOR signaling pathway, thereby influencing the expression of downstream glycolysis-related proteins.

## Discussion

Presently, much of the research concerning B7-H3 revolves around its interactions with immune cell surface ligands. Nevertheless, emerging evidence suggests an additional role for B7-H3 in promoting tumor progression through its impact on tumor glycolysis 2, 20, 23, 38, 39.

Previous findings underscore the existence of a metabolic competition occurring between cancer cells and immune cells, with various immune cell subsets being closely regulated by specific metabolic programs 40, 41. A significant glucose competition between tumor cells and CD8^+^ T cells is evident, and B7-H3 plays a pivotal role in augmenting tumor cells' capacity to compete for glucose resources, resulting in the suppression of CD8^+^ T cell glycolysis.

In our research, we discovered that B7-H3 enhances glycolysis in tumor cells while simultaneously inhibiting glycolysis in CD8^+^ T cells. Further examination of intracellular levels of IFN-γ, granzyme B, PD-1, and Tim-3 in tumor-infiltrating lymphocytes illuminated changes in the immune functionality of CD8^+^ T cells. Notably, CD8^+^ T cells experiencing suppressed glycolysis demonstrated diminished immune functions and an increased state of exhaustion. This aligns with existing literature indicating the compromised effector functions of CD8^+^ T cells within the tumor microenvironment 35. Additionally, we observed that the glycolysis inhibition could be counteracted by adding exogenous glucose. Moreover, our *in vitro* mouse transplantation model showed that tumors with enhanced glycolysis exhibited increased tumor-forming capabilities. These findings lead us to tentatively conclude that B7-H3 amplifies the Warburg effect in tumor cells while simultaneously suppressing CD8^+^ T cell glycolysis, thereby dampening their immune responses. It is essential, however, to acknowledge that further mechanisms may be involved, requiring in-depth exploration for a comprehensive understanding.

Previous research has demonstrated B7-H3's role in promoting the translation of HIF-1α via the PI3K/Akt/mTOR pathway, thereby increasing glycolysis in OSCC.^24^. In our study, we assessed the effect of B7-H3 on the expression of PI3K, Akt, and mTOR proteins in SCC7 and B16-F10 cells. We found that the overall expression levels of these proteins remained unchanged across different B7-H3 expression levels. However, overexpression of B7-H3 markedly increased the phosphorylation of PI3K, Akt, and mTOR proteins. This activation led to enhanced expression of HIF-1α and its downstream glycolysis-related proteins, GLUT1 and PFKFB3. The exact mechanism by which B7-H3 influences phosphorylation within the PI3K/Akt/mTOR pathway warrants further investigation.

While substantial research has delved into the oncogenic properties of B7-H3, our investigation pivots towards a nuanced understanding of its involvement in the metabolic reconfiguration of tumors and the consequential ramifications on immune functionalities. This exploration extends beyond the conventional interplay of direct recognition and interaction between tumor cells and immune constituents. We uncover that fluctuations in B7-H3 expression intricately modulate the metabolic pathways of CD8^+^ T cells, with profound implications for their operational efficiency. Our research demonstrates that different expression levels of B7-H3 can indirectly influence the metabolic processes of CD8^+^ T cells, thereby impacting their functionality. This interplay between B7-H3 expression and T cell metabolism, particularly in the context of cancer progression, is a dimension that has not been adequately explored in previous studies. It underscores a crucial biology how metabolic alterations driven by tumor-expressed molecules like B7-H3 can subvert the immune system's ability to combat cancer. This intriguing observation may help elucidate the reason why B7-H3, despite possessing immune co-stimulatory functions, can still promote cancer and how it facilitates a tumor-supportive microenvironment. Specifically, by adjusting the metabolic landscape, B7-H3 may create a nutrient-deprived environment for CD8^+^ T cells, thus impairing their ability to function effectively against tumor cells. In essence, our study expands the understanding of B7-H3's role in cancer progression, highlighting its significance in the metabolic reprogramming of tumors and its indirect effects on immune cell functionality. By delineating the multifaceted strategies employed by tumors to evade immune responses, including the manipulation of metabolic pathways, our research underscores the importance of targeting metabolic processes in the development of novel cancer therapeutics. The implications of these findings are profound, suggesting that disrupting the metabolic interplay orchestrated by B7-H3 could offer a promising avenue to enhance anti-tumor immunity and impede cancer progression.

However, we must acknowledge that our research on CD8^+^ T cells is not exhaustive. Future studies will require a multifaceted approach to comprehensively validate the impact of glycolytic inhibition on CD8^+^ T cell functionality. Additionally, while we observed that the introduction of exogenous glucose could facilitate the restoration of CD8^+^ T cell functions under glycolytic inhibition, we did not verify this in the presence of tumor cells nor did we conduct subsequent animal experiments. These aspects represent critical areas for future research to address and refine. Further, it is pertinent to acknowledge that current research has suggested the existence of two primary forms of B7-H3 in cells: one as a transmembrane protein on the cell membrane and the other as a soluble form in the cytoplasm 42. For instance, pancreatic cancer cells can release soluble B7-H3 into the extracellular matrix, and B7-H3 has also been identified in extracellular vesicles derived from neuroblastoma cell lines. These findings suggest that B7-H3 may facilitate cellular interactions in tumor dissemination and promote tumor development 43. Notably, these distinct regions and modalities of B7-H3 may possess varying roles and mechanisms, a facet that our current study did not encompass. Thus, it becomes imperative for future research to delve into the relationship between different modalities of B7-H3 and tumor development in OSCC and melanoma.

Subsequent investigations could also explore whether other metabolic pathways contribute to the competition between tumor cells and immune cells, and ascertain whether B7-H3 influences tumor development through other metabolic pathways or modes. Additionally, exploring the potential synergistic effects of B7-H3 inhibitors in combination with other immunotherapeutic drugs to enhance immune cell suppression could be a promising avenue for future studies. Such research endeavors may pave the way for novel therapeutic strategies aimed at mitigating tumor immune evasion and improving overall cancer treatment outcomes.

## Figures and Tables

**Table 1:** The expression level of B7-H3 in 57 clinical OSCC specimens.

No.	Location	Gender	Age	Alcohol	Cigarette	Tumor stage	Pathological classification	Recurrence	Lymph node metastasis	CD276
#1	Tongue	Female	51	N	N	IV	I	N	Y	4.5
#2	Tongue	Male	56	Y	Y	IV	II	N	N	2.5
#3	Gingiva	Female	76	N	N	IV	II	N	Y	1.5
#4	Tongue	Female	30	N	N	III	I	N	N	4
#5	Buccal	Male	41	Y	Y	IV	II	N	N	4.5
#6	Tongue	Male	41	N	Y	II	II	N	Y	4
#7	Tongue	Female	64	N	N	III	II	N	Y	5.5
#8	Tongue	Male	53	Y	N	III	II	N	Y	4.5
#9	Tongue	Female	66	N	N	III	I	N	Y	6
#10	Buccal	Male	69	Y	Y	IV	I	N	Y	4
#11	Oral floor	Male	71	Y	Y	IV	I	N	Y	6.25
#12	Buccal	Male	76	N	N	II	II	N	N	2.5
#13	Tongue	Female	49	N	N	II	I	N	N	4
#14	Tongue	Female	52	N	N	I	I	N	N	2
#15	Oral floor	Male	63	Y	Y	IV	III	N	Y	6.25
#16	Buccal	Female	54	N	N	III	I	N	Y	5.75
#17	Buccal	Female	70	N	N	III	II	N	Y	5
#18	Oral floor	Female	55	N	N	IV	II	Y	Y	5
#19	Oral floor	Male	47	Y	Y	IV	I	N	Y	6.75
#20	Other	Female	53	N	N	III	I	Y	N	2.75
#21	Tongue	Male	66	Y	N	III	II	N	Y	2
#22	Tongue	Male	50	Y	Y	III	I	Y	N	2
#23	Gingiva	Female	68	N	N	II	I	N	N	3.25
#24	Buccal	Male	59	Y	Y	III	I	N	N	4
#25	Other	Male	40	Y	Y	IV	I	N	Y	4.5
#26	Buccal	Male	68	Y	N	III	I	N	Y	5.5
#27	Buccal	Male	72	N	N	II	II	N	N	3.75
#28	Gingiva	Female	78	N	N	IV	I	N	Y	4.5
#29	Gingiva	Female	54	N	N	IV	I	N	Y	5.5
#30	Buccal	Female	56	N	N	IV	I	N	Y	7.5
#31	Buccal	Male	57	N	N	IV	I	N	Y	7.5
#32	Buccal	Female	77	N	N	IV	II	N	Y	8.25
#33	Gingiva	Male	74	N	N	IV	II	N	Y	6.25
#34	Other	Male	61	Y	Y	IV	III	N	Y	4.5
#35	Other	Male	69	Y	Y	IV	II	N	Y	5.5
#36	Buccal	Female	76	Y	N	IV	I	N	Y	6
#37	Oral floor	Male	64	Y	Y	III	II	N	Y	7.5
#38	Tongue	Female	32	N	Y	III	II	N	Y	6.75
#39	Tongue	Male	44	Y	Y	IV	I	N	N	5
#40	Tongue	Female	33	Y	Y	III	II	N	Y	7.5
#41	Tongue	Female	56	N	N	II	I	Y	N	3.5
#42	Tongue	Female	36	Y	Y	II	I	N	N	3
#43	Other	Male	62	N	Y	III	II	N	N	5
#44	Buccal	Female	65	N	N	IV	II	N	N	5
#45	Tongue	Male	37	N	Y	III	I	N	N	2.5
#46	Oral floor	Male	72	Y	Y	IV	III	Y	N	6
#47	Buccal	Female	76	N	N	IV	I	N	N	4.5
#48	Other	Male	61	N	Y	II	I	N	N	1.75
#49	Other	Male	76	N	Y	III	III	Y	N	4.5
#50	Tongue	Male	73	Y	Y	III	I	N	N	3.25
#51	Tongue	Male	63	Y	Y	III	I	N	N	3
#52	Other	Male	49	Y	Y	IV	I	N	Y	5
#53	Buccal	Male	67	N	N	II	I	N	N	4.25
#54	Other	Male	59	Y	Y	IV	II	N	Y	5
#55	Tongue	Male	69	Y	Y	IV	II	N	Y	6.25
#56	Tongue	Female	55	N	N	IV	II	N	Y	5.5
#57	Tongue	Female	61	N	N	II	I	Y	N	4.25

**Table 2:** The relationship between the expression level of B7-H3 and clinicopathological parameters in 57 OSCC specimens.

Parameters	Case NO.		CD276 IHC Score	P Value
Age	>50	44	4.71±1.63	0.797
	<=50	13	4.58±1.65	
Gender	male	32	4.55±1.58	0.514
	female	25	4.84±1.68	
Alcohol consumption	Y	25	4.81±1.58	0.435
	N	32	4.58±1.66	
Cigarette smoking	Y	27	4.69±1.63	0.433
	N	30	4.67±1.63	
Location	tongue	22	4.15±1.60	0.023^a^
	buccal	5	5.20±1.58	
	gingiva	9	4.20±1.88	
	oral floor	15	6.29±0.83	
	other sites	6	4.27±1.22	
Tumor stage	Tis-T2	11	3.30±0.89	0.001
	T3-T4	46	5.01±1.58	
Lymphnode metastasis	Y	32	5.50±1.48	0.000
	N	25	3.63±1.10	
Recurrence	Y	7	4.78±1.64	0.239
	N	50	4.00±1.36	

a. Using Bonferroni analysis, the expression level of B7-H3 differs at the location of tongue and oral floor
